# Synthesis and Chemistry of Organic Geminal Di- and Triazides

**DOI:** 10.3390/molecules201119675

**Published:** 2015-11-06

**Authors:** Andreas P. Häring, Stefan F. Kirsch

**Affiliations:** Organic Chemistry, Bergische Universität Wuppertal, Gaußstraße 20, 42119 Wuppertal, Germany; haering@uni-wuppertal.de

**Keywords:** diazide, triazide, carbonyl diazide, diazidomethane, triazidomethane, tetraazido-methane, diazidation, azidation

## Abstract

This review recapitulates all available literature dealing with the synthesis and reactivity of geminal organic di- and triazides. These compound classes are, to a large extent, unexplored despite their promising chemical properties and their simple preparation. In addition, the chemistry of carbonyl diazide (**2**) and tetraazidomethane (**105**) is described in separate sections.

## 1. Introduction

In contemporary organic chemistry, the azide group is one of the functional groups that has received the greatest increase in attention over the last decades [1,2]. In particular, the use of its orthogonal reactivity to other functional groups has led to a broad range of applications. Thus, cycloadditions with alkynes are routinely used in chemical biology [3,4,5,6,7] and material sciences [8,9,10]. While, in this context organic molecules with one azide group are widely accepted as standard entities, small molecules with two or more azide groups attached to the same carbon atom are quite uncommon. This lack of interest is at least a bit surprising, given the fact that reports on carbonyl diazide date back to 1894, and ethyl 2,2-diazidoacetate was described first in 1908 (see below). However, the supposedly hazardous and explosive character of geminal diazides might have hampered research efforts in the field, and up to now the chemical scope of geminal di- and triazides has not been studied in full detail.

In the following review, we summarize the reports on the syntheses of organic geminal di- and triazides as well as of carbonyl diazide and tetraazidomethane. We also present an overview on the reactivity of these compounds revealed so far.

## 2. Carbonyl Diazide

**Scheme 1:**
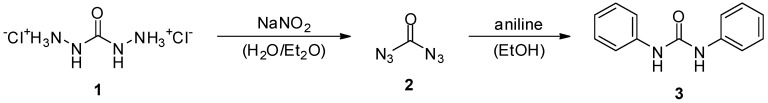
Synthesis of carbonyl diazide (**2**) from carbonyl dihydrazide dihydrochloride (**1**) and transformation to diphenylurea (**3**).

The first record of carbonyl diazide (**2**) dates back to 1894, when Curtius and Heidenreich reported its synthesis and some reactions performed for analytical purposes [11,12]. By treating an aqueous solution of carbonyl dihydrazide dihydrochloride (**1**) with two equivalents of sodium nitrite at low temperature the crystalline carbonyl diazide (**2**) was obtained (Scheme 1). The compound was described as very volatile and highly explosive on impact or when exposed to bright light, and it was noted that carbonyl diazide (**2**) reacts like phosgene. Since a direct confirmation for its structure was not possible at this time, the diazide **2** was saponificated and an argentometric determination of the azide content was performed. Moreover, treatment with aniline led to the corresponding urea **3**, a known compound at this time. Elemental analysis of carbonyl diazide (**2**) resulted in somewhat imprecise figures, which was believed to be caused by the volatility and the small amounts of substance which were used due to its hazardousness. In 1924, 30 years later, Kesting pointed out, that these inaccuracies may have arisen from impurities such as the symmetrical hydrazine-1,2-dicarbonyl azide, which is formed as a by-product during the generation of carbonyl diazide [13].

**Scheme 2:**
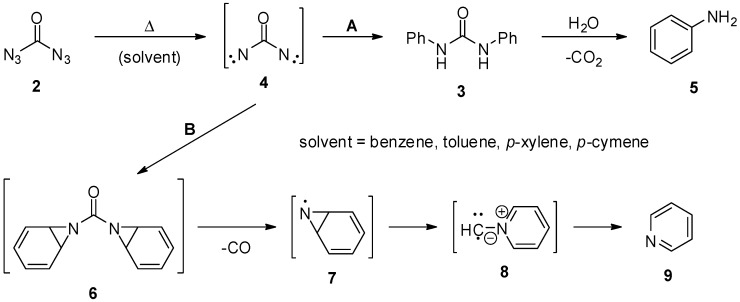
Thermal reactions of carbonyl diazide (**2**) in aromatic hydrocarbons.

In a detailed examination, Curtius and Bertho then tried to elucidate the outcome of the thermal reactions between carbonyl diazide (**2**) and aromatic hydrocarbons [14]. Unfortunately, the result of these investigations is hardly conclusive: the original experiment basically consisted of heating carbonyl diazide (**2**) (prepared *in situ* from carbonyl dihydrazide dihydrochloride (**1**), Scheme 1) in several aromatic hydrocarbons, and the reaction mixtures were finally analyzed for their constituents. Surprisingly, aromatic amines **5** and pyridines **9** were identified as the major non-volatile products in all cases. Due to the concomitant formation of many unidentified products, the loss of several volatile compounds, and the fact that carbonyl diazide (**2**) was prepared *in situ*, the authors hesitated to give yields for the formation of **5** and **9**. Nevertheless, the simple existence of amines **5** and pyridines **9** in the reaction mixtures requires an interesting reaction mechanism. To this end, Curtius and Bertho proposed two competitive mechanisms (Scheme 2): the formation of aromatic amines (**5**, path A) is explained by the thermal loss of two molecules of nitrogen from carbonyl diazide (**2**) forming a dinitrene **4**. This very reactive species **4** was believed to undergo an insertion into the carbon-hydrogen bond of the aromatic hydrocarbons. Under high temperatures, and in the presence of water, the resulting urea derivatives **3** are then hydrolyzed to give aromatic amines **5** and carbon dioxide, which was indeed identified when analyzing the volatile products. The formation of pyridines (**9**, path B) was less clear and requires not only a rearrangement of the aromatic core, but also the cleavage of a methine group. In this case, the dinitrene **4** was believed to form an aziridine **6**. After loss of carbon monoxide, which was also detected in the gas phase, this aziridine **6** might rearrange in an unspecified manner to give the pyridine core. The authors speculated on the existence of intermediates **7** and **8**. The fate of the lost carbon atom, however, could not be clarified. Nevertheless, this early work is an impressive example for the reactive power of carbonyl diazide (**2**), a compound that was then forgotten for almost a century.

**Scheme 3:**
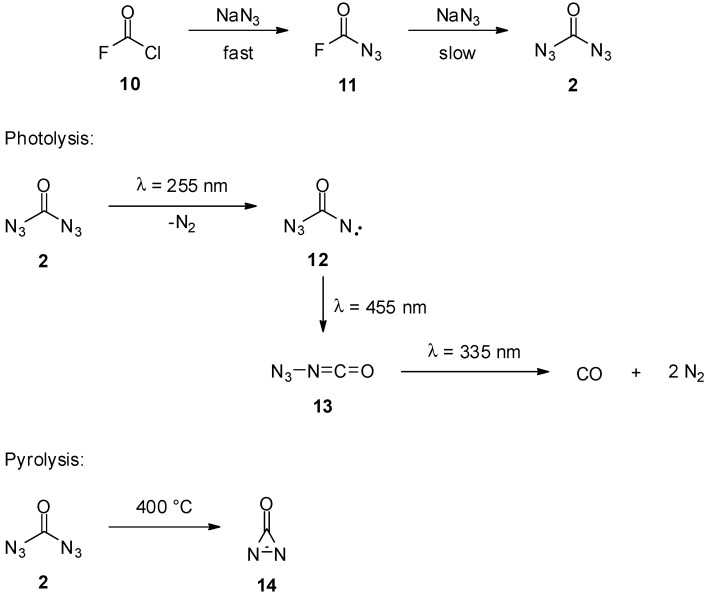
Synthesis, photolysis and pyrolysis of carbonyl diazide (**2**).

The “rediscovery” of carbonyl diazide (**2**) by Willner *et al.* in 2010 resulted in a detailed description of its structure and physical properties [15]. By reacting sodium azide with fluoro- carbonyl chloride (**10**) in a sealed glass ampule, they were able to get pure carbonyl diazide (**2**) in small quantities as a white solid via fluorocarbonyl azide (**11**) as intermediate after fractionated condensation (Scheme 3). From this sample the authors obtained infrared, Raman and UV/VIS spectra in the solid state, as well as in the gaseous phase. Furthermore the structure was confirmed by X-ray diffraction. Of note, all experimental data were in good agreement with several independent calculations [15,16,17,18,19]. Very interesting from a preparative point of view, carbonyl diazide (**2**) is thermally stable with a defined melting point of 16 °C, and it shows a high impact sensitivity. Regarding the reactivity of carbonyl diazide (**2**), the photolysis [20] and the thermal decomposition [19,21,22] were investigated by means of infrared spectroscopy and theoretical calculations (Scheme 3). The irradiation of matrix-isolated carbonyl diazide (**2**) with UV-light (255 nm) causes the loss of molecular nitrogen and the formation of azido carbonyl nitrene **12**, which rearranges under visible light (455 nm) to give azido isocyanate (**13**). By further irradiation of azido isocyanate (**13**) with UV-light (335 nm), carbon monoxide and molecular nitrogen are obtained as final products. The pyrolysis of carbonyl diazide (**2**) was performed by passing diluted gaseous carbonyl diazide (**2**) through a glass tube heated to 400 °C and trapping of the products. Via infrared spectroscopy, metastable diazirinone (**14**) was identified as the major pyrolysis product.

**Scheme 4:**
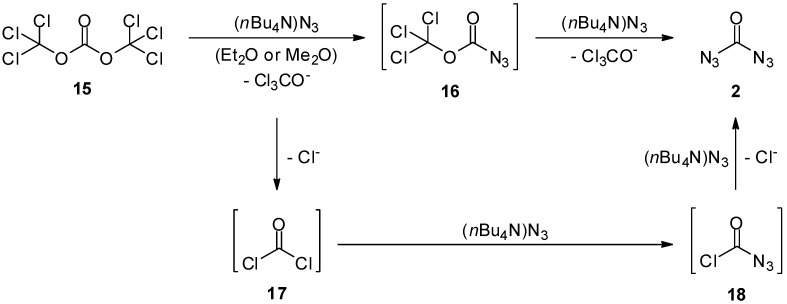
Carbonyl diazide (**2**) synthesis from triphosgene (**15**).

An alternative synthesis of carbonyl diazide (**2**) was presented by Stanton *et al.* [17]. Starting from triphosgene (**15**), carbonyl diazide (**2**) was generated through double substitution with tetrabutylammonium azide (Scheme 4). The substitutions occur on different reaction intermediates (**16**, **17**, **18**), which all lead to the same target. The product **2** was isolated by simple filtration of the ammonium salts followed by evaporation of the solvent. The infrared spectra obtained from carbonyl diazide (**2**), which was prepared this way, were identical to those from the abovementioned method by Willner *et al.* [15]. The major advantage of the protocol developed by Stanton *et al.* is the commercial availability of all the starting materials. The use of highly toxic fluorocarbonyl chloride (**10**) can be avoided, and the reaction can be carried out in solution at room temperature under experimentally simple conditions.

Also noteworthy is the formation of carbonyl diazide (**2**) via hydrolysis of tetraazidomethane (**105**) (Scheme 22, below) [23]. As tetraazidomethane (**105**) is not an easily available starting material, this transformation cannot be considered useful for preparative purposes; the generation of tetraazidomethane (**105**) will be discussed below in greater detail.

## 3. Geminal Diazides

### 3.1. Geminal Aliphatic Diazides

#### 3.1.1. Synthesis

**Scheme 5:**
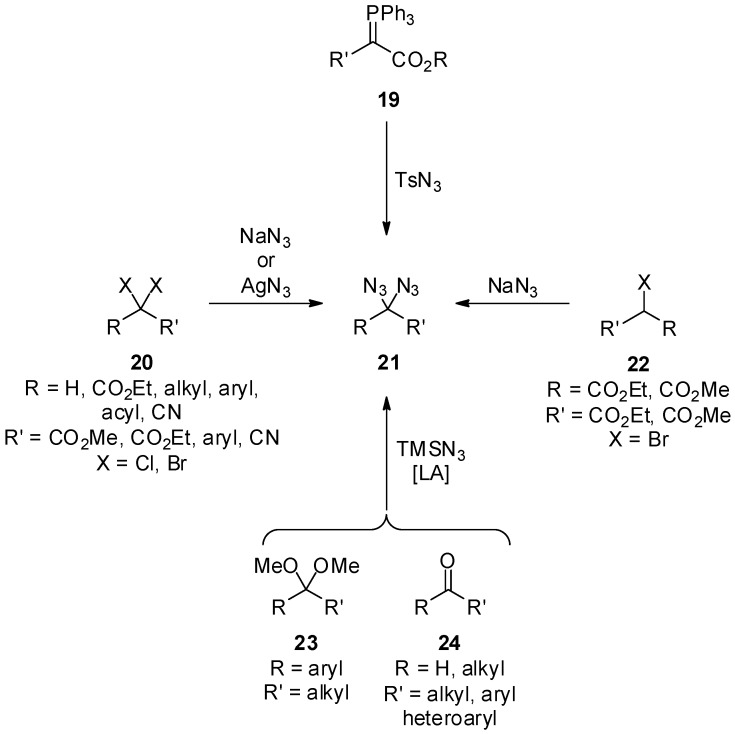
Classic methods for the preparation of geminal aliphatic diazides **21**.

The first method reported for the synthesis of geminal diazides **21** is the direct substitution of geminal dihalides **20** with sodium azide (Scheme 5), and this remains unquestionably one of the most reliable and simplest methods until today. In 1908, Forster *et al.* succeeded in isolating ethyl 2,2-diazidoacetate [24]. In their seminal report, the authors already mentioned the hazardous character of this compound class, which mainly results from the risk of explosion [25,26]. In the subsequent decades, a series of α,α-diazidoesters [24,27,28], α,α-diazido-β-ketoesters [29,30], α,α-diazidoketones [31], α,α-diazido-β-ketolactams [32], 2,2-diazidomalonates [33], heterocyclic diazides [32,34,35,36] and benzylic diazides [25,37,38,39,40,41] were prepared using standard halide substitution chemistry. As an alternative azide source, an insoluble polymeric ammonium azide resin was also employed [42,43].

Alternatively, diazidomalonates are available via the disproportionation of alkyl 2-bromomalonates **22** (Scheme 5) [44]. This reaction is of less use, since half the amount of the alkyl 2-bromomalonate **22** is lost as alkyl malonate. However, as no monoazide is formed, the reaction gives access to 2,2-diazidomalonates of high purity [45,46].

Electrophilic azide sources can also be used for the diazidation task. For example, phosphorous ylides **19** were shown to give geminal diazides **21** when reacted with tosyl azide (Scheme 4) [27]. Likewise, Evans *et al.* observed geminal diazides **21** as minor by-products when the azidation of ester enolates with 2,4,6-triisopropylbenzenesulfonyl azide was investigated [47].

Acetals and ketones can be easily transformed into geminal aliphatic diazides **21** with trimethylsilyl azide (Scheme 5) [48]. This reaction is typically catalyzed with strong Lewis acids (LA) like tin(IV) chloride. In principle, the transformation can be understood best as a simple transacetalization. Ketones [49,50] or aldehydes [50,51] were also employed as starting materials for the diazide generation; in these cases, tin(II) chloride was used as the preferred catalyst. The method was further expanded to not only aromatic and aliphatic aldehydes, but also to heterocyclic aldehydes [52]. Simple addition of catalytic amounts of sodium azide in combination with a crown ether was shown to further promote this reaction [50]. Another variant showed that, by using aluminium(III) chloride or titanium(IV) chloride as catalysts, aldehydes can directly be converted to geminal diazides **21** with sodium azide instead of trimethylsilyl azide [53,54].

**Scheme 6:**
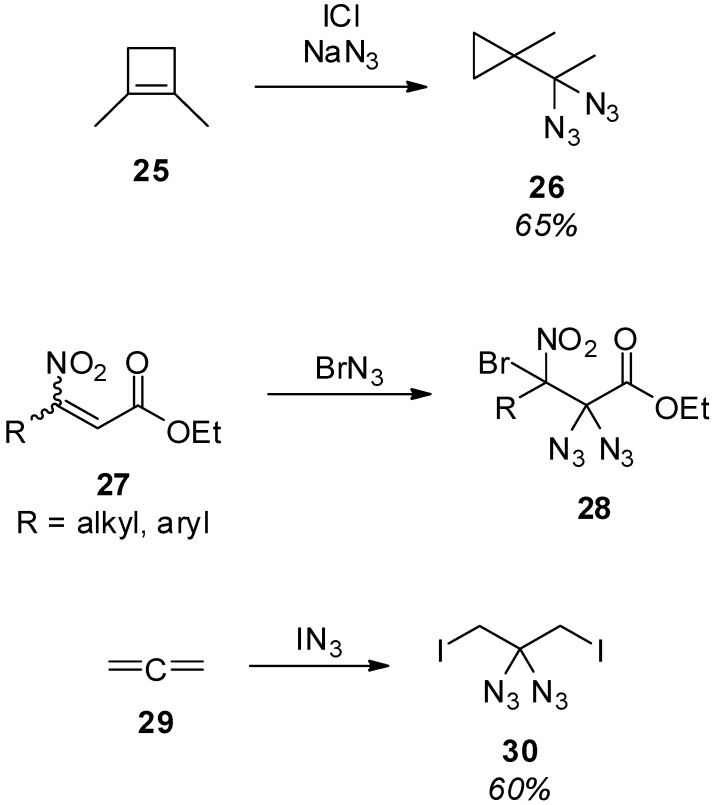
Diazidation with iodine and bromine azide.

An unexpected formation of a geminal diazide **26** was reported by Swern and O’Hare (Scheme 6) [55]. When the reactivity of 1,2-dimethylcyclobutene (**25**) was studied, the use of an excess of iodine chloride and sodium azide led to a ring contraction and diazidation of the exocyclic carbon atom. However, only the single example shown in Scheme 6 was presented, and the mechanism and scope of this reaction was not surveyed. Related to the use of iodine chloride and sodium azide, also bromine azide was used to transform, for example, nitroolefins into geminal diazides **28** via an addition-elimination-addition sequence (Scheme 6) [56]. Furthermore, allene (**29**) was successfully converted to geminal diazide **30** with iodine azide [57].

**Scheme 7:**
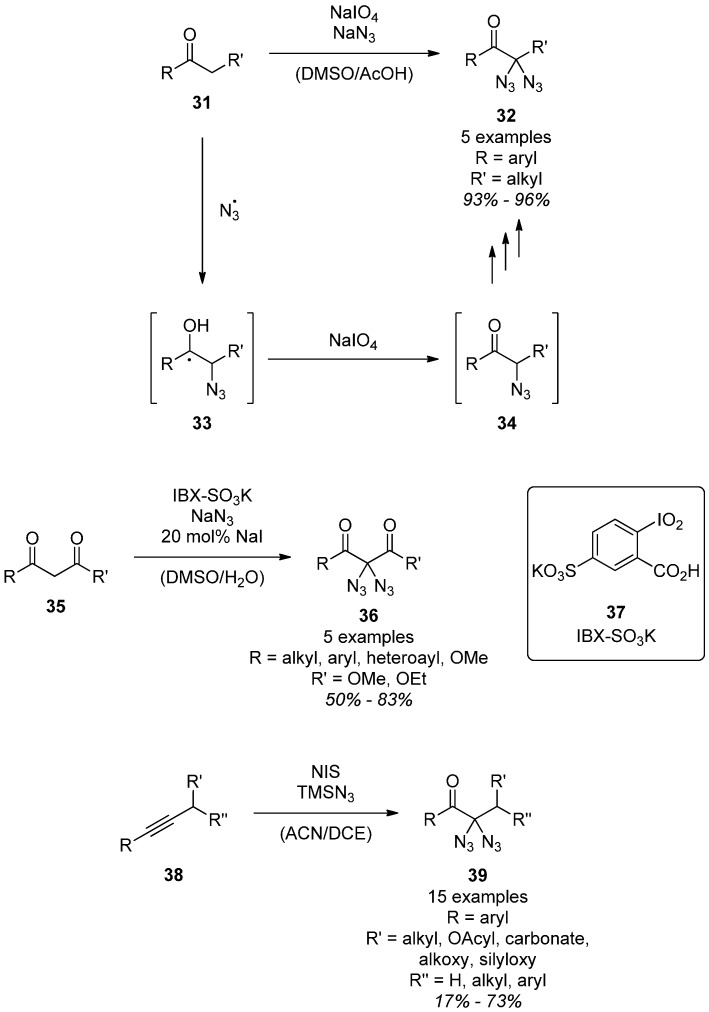
Oxidative diazidation methods.

The most recent diazidation-methods allow for the direct α-diazidation of benzylic alcohols [58], ketones [58,59], internal olefins [59] and alkynes [60] as well as of malonates and β-ketoesters [61] under oxidative conditions. In all cases the products are α,α-diazidocarbonyl compounds. The oxidizing agents of choice are sodium periodate [58], IBX-SO_3_K (**37**) [59,61] and *N*-iodosuccinimide [60]. The first one, sodium periodate, gave α,α-diazidoketones **32** in excellent yields when applied to ketones **31** in combination with sodium azide (Scheme 7) [58]. A proposed mechanism for this transformation includes the one-electron-oxidation of an azide ion, followed by addition to the enol tautomer of a benzylic ketone **31**. Further oxidation of the resulting benzylic radical **33** then gives the α-azidoketone **34**, which can undergo this cycle one more time to furnish α-diazidoketones **32**. The monoazide **34** was never found under the conditions, even when the reaction was carried out with only one equivalent of sodium azide. From this result one can deduce that the second azidation cycle has to be significantly faster than the first one.

Another very mild and experimentally simple diazidation protocol developed by Kirsch *et al.* uses IBX-SO_3_K (**37**) [62] in combination with catalytic amounts of sodium iodide and an excess of sodium azide at room temperature (Scheme 7) [61]. By using this method, a series of malonates and β-ketoesters **35** were successfully converted into their corresponding diazides **36**. The fundamental mechanism is still under investigation, although it is plausible that iodination and subsequent substitution takes place. This method was further expanded not only to ketones, but also to internal olefins as starting materials [59]: Via a one-pot procedure the olefins were first reacted with IBX and *N*-iodosuccinimide to give α-iodoketones, which then were diazidated using IBX-SO_3_K (**37**) and sodium azide.

With internal alkynes **38** as starting materials and trimethylsilyl azide, Yanada *et al.* developed a highly useful protocol for the synthesis of diazides (Scheme 7) [60]. The presence of moisture and oxygen from the air is necessary for the reaction to proceed smoothly, but the exact role of water and oxygen was not clarified. On the other hand, it was observed that additional water in the reaction mixture led to markedly decreased yields, probably due to the fast hydrolysis of trimethylsilyl azide. The origin of the carbonyl oxygen is also unclear. As a possible mechanism, the twofold addition of iodine azide in a regioselective manner followed by the hydrolysis of the resulting geminal diiodide is assumed.

#### 3.1.2. Reactivity

**Scheme 8:**
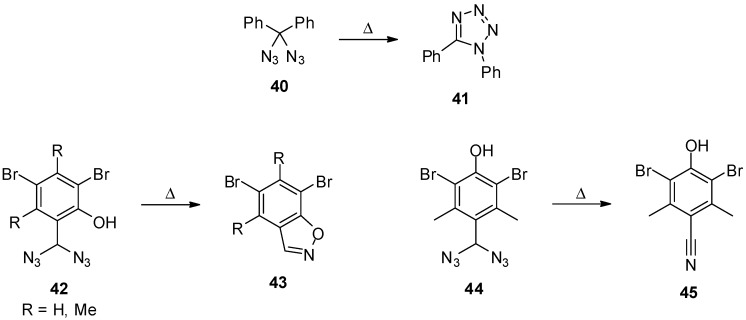
Thermal reactions of benzylic geminal diazides.

There is only a limited number of publications that focus on the reactivity of geminal aliphatic diazides **21**. In most reports, diazides **21** are only mentioned as by-products or proposed intermediates [31,34,35,47,63,64,65,66,67,68,69,70,71,72,73,74,75,76,77,78,79]. A few reports discuss thermodynamic and kinetic influences of the azido group on the stability of carbocations [80,81,82,83] and calculated structures of geminal diazides [84]. In particular, the early reports about these substances and their reactivity are somewhat lacking in data due to the lack of analytical tools. Nevertheless, these early reports contain highly valuable hints about the outstanding reactions of geminal aliphatic diazides **21**, even if the reaction products could not be identified without a doubt. For example, Schroeter already postulated in 1909 that the thermal decomposition of diazidodiphenylmethane (**40**) leads to the tetrazole derivative **41** (Scheme 8) [37], a degradation that was unequivocally confirmed some years later [39]. In contrast, Lindemann and Mühlhaus found isoxazoles **43** and nitriles **45** when they studied the thermal behavior of *ortho*- (**42**) and *para*-hydroxybenzylic diazides **44** [38]. Additionally, they obtained the corresponding benzaldehydes of the diazides **42** and **44**, when the thermolysis was performed in ethanol. The latter observation could probably be explained by simple hydrolysis due to water in the solvent. Unfortunately, the available analytical data are not sufficient for an all-encompassing theory on the reaction mechanisms. However, a number of more recent publications also describe protocols for the synthesis of benzonitriles by use of reagents such as sodium azide or trimethylsilyl azide [36,53,54,85,86], and mostly benzylic diazides generated *in situ* are believed to be intermediates or competitive products.

**Scheme 9:**
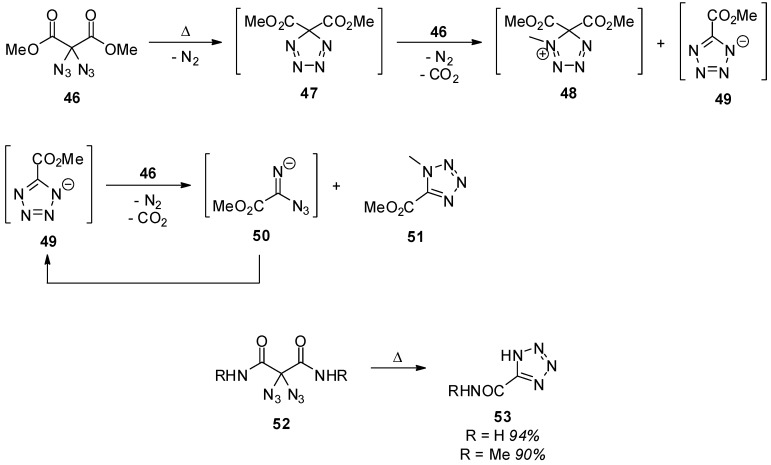
Thermal reaction of diazidomalonates **46** and –malonamides **52**.

The reaction of geminal diazides **21** under elevated temperatures has remained a major focus for many years. For example, methyl 2,2-diazidomalonate (**46**) and derivatives **52** thereof were thermally converted into tetrazoles **51** and **53** (Scheme 9) [87]. The originally proposed mechanism consisted of the insertion of a nitrene into the oxygen-carbon bond followed by expulsion of carbon dioxide. It is noteworthy that this decomposition pathway differs significantly from the photochemical decomposition of geminal diazides as discussed below, and it was possible to achieve a good control over the formation of differently substituted tetrazoles depending on whether the reaction is carried out thermally or under irradiation with light [88]. Several additional examples for tetrazole formation through thermal decomposition of geminal aliphatic diazides provide evidence for the nitrene insertion mechanism [32,89,90]. However, a crossover experiment with isotope-labeled starting materials showed product mixtures [46] indicating that, at some stage, an intermolecular mechanism takes place. Based on these results, the mechanistic picture was refined, and an anionic chain mechanism was suggested as outlined in Scheme 9. It was also found that, instead of elevated temperatures, the use of catalytic amounts of Lewis acids like tin(IV) chloride or zinc(II) chloride can lead to the formation of tetrazoles [48,49,51].

**Scheme 10:**
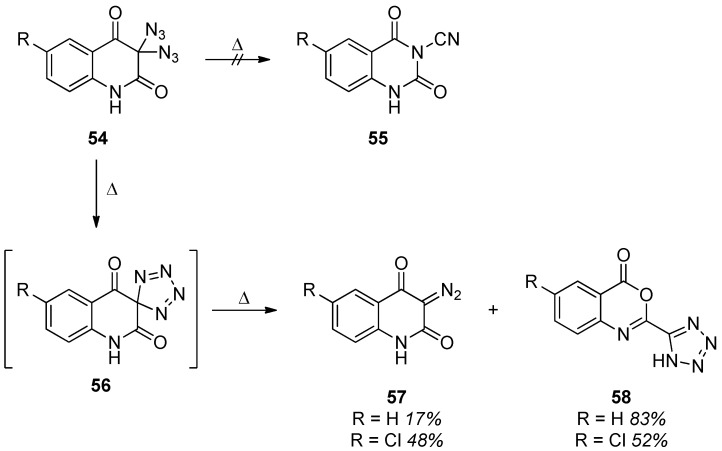
Thermal reaction of 3,3-diazido-2,4-dioxoquinolines **54**.

When the thermal behavior of 3,3-diazido-2,4-dioxoquinolines **54** was studied, it was originally expected that tetrazole intermediates can be further converted into *N*-cyano moieties **55** (Scheme 10) [32]. Although this assumption turned out to be wrong, the data suggest that the isomeric diazo compounds **57** can be generated under the conditions [91]. Besides the diazo compound **57**, the formation of a tetrazole **58** was also observed. It was proposed that both **57** and **58** were formed through tetrazole **56** as the common intermediate although the mechanistic pathway was not investigated at all. Moreover, other reports also postulate the occurrence of diazo species in the thermal degradation of a geminal diazide [92].

**Scheme 11:**
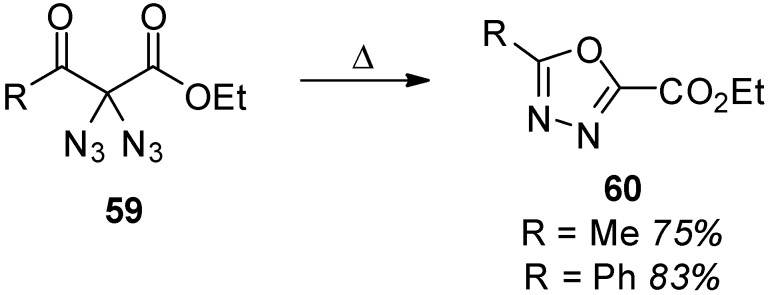
Synthesis of 1,3,4-oxadiazoles **60** from α,α -diazido-β-ketoesters **59**.

Ogilvie and Rank described the synthesis of 1,3,4-oxadiazoles **60** through heating of α,α-diazido-β-ketoesters **59** (Scheme 11) [30]. The product formation was again explained with a mechanism based on nitrene formation followed by rearrangement. The structure of the 1,3,4-oxadiazoles **60** was unequivocally confirmed by comparison with samples that were synthesized through alternative routes.

**Scheme 12:**
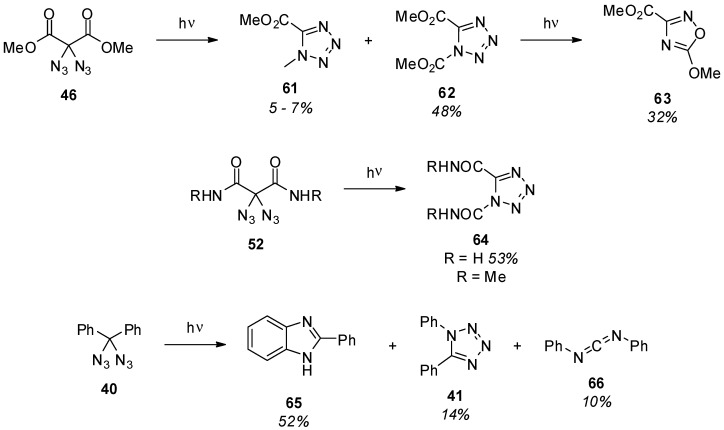
Photochemical decomposition of methyl 2,2-diazidomalonate (**46**), 2,2-diazidomalonamides **52** and diazidodiphenylmethane (**40**).

The photolysis of methyl 2,2-diazidomalonate (**46**) [45], 2,2-diazidomalonamides **52** [87] and diazidodiphenylmethane (**40**) [93] was studied by Moriarty *et al.* (Scheme 12). A range of different products were obtained, suggesting that more than one possible reaction pathway is involved. Several mechanistic aspects were presented including the formation of nitrene intermediates, which was also supported by EPR studies [40]. However, a conclusive mechanism explaining all the observations regarding diazide photolysis is still not available. From a synthetic point of view, tetrazole **62** was obtained as the major product upon irradiation of **46**. Further irradiation of this intermediate **62** then leads to nitrogen loss, and methyl 5-methoxy-1,2,4-oxadiazole-3-carboxylate (**63**) is obtained. 2,2-Diazidomalonamides **52** reacted in a similar way leading to tetrazoles **64**. Diazidodiphenylmethane (**40**), on the other hand, shows a fully different product distribution (**65**, **41** and **66**). More recent studies on the photochemistry of geminal benzylic diazides aided by DFT-calculations and isotope-labeling experiments also came to the result that, most likely, multiple reaction pathways are involved [43].

**Scheme 13:**
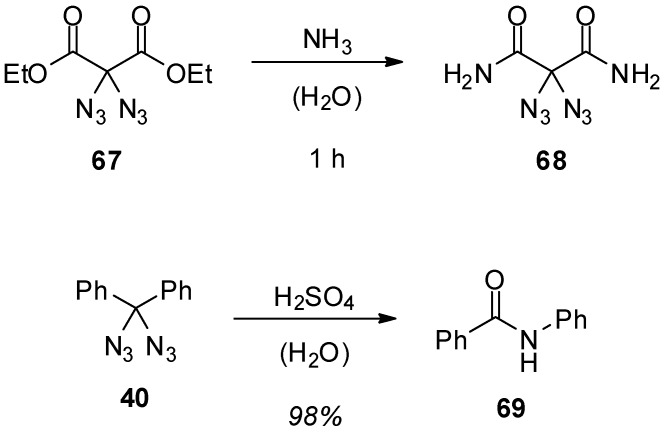
Simple intermolecular reactions of geminal diazides.

Besides intramolecular reactions of geminal aliphatic diazides, only a few intermolecular reactions are known. The first one is the formation of 2,2-diazidomalonamide (**68**) (Scheme 13) [33]: In the presence of ammonia, ethyl 2,2-diazidomalonate (**67**) is easily transformed into its amide **68**. The azido groups are not directly involved in this reaction, but they seem to have a strong activating effect since with simple ethyl malonate the amide formation is significantly slower. Another intriguing reaction is the conversion of diazidodiphenylmethane (**40**) into *N*-phenylbenzamide (**69**) under the influence of strong acids [39]. This reaction is explained best by a sequence consisting of hydrolysis of the diazide **40**, the formation of a nitrene-intermediate due to the loss of molecular nitrogen and subsequent rearrangement.

**Scheme 14:**
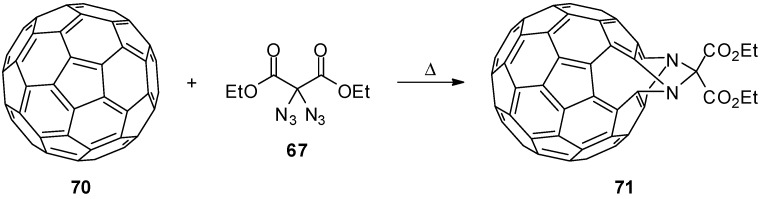
Functionalisation of C_60_-fullerene (**70**) with ethyl 2,2-diazidomalonate (**67**).

In an impressive report, the potential of geminal diazides as nitrene precursors was highlighted [94]: C_60_-Fullerene (**70**) was heated together with ethyl 2,2-diazidomalonate (**67**) (Scheme 14). The main product of this fullerene functionalization was a doubly bridged fulleroid **71**.

### 3.2. Geminal Vinyl Diazides

**Scheme 15:**
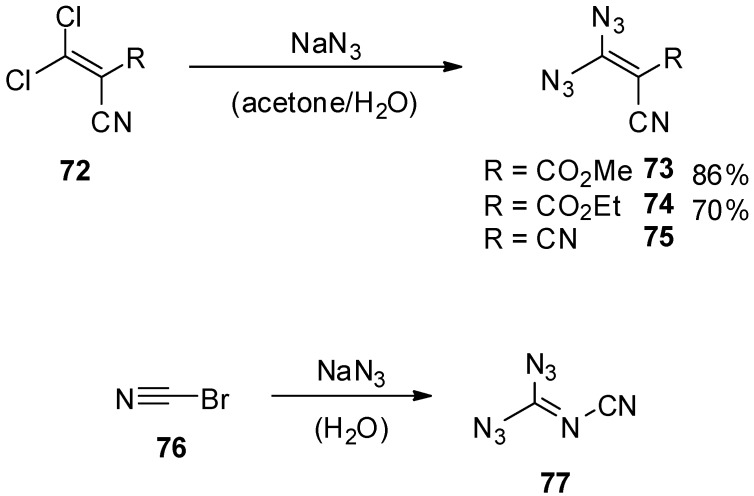
Synthesis of all known geminal vinyl diazides.

The number of reports describing geminal vinyl diazides is, as one might expect, very small. In fact, only three molecules of this class (compounds **73**–**75**) were synthesized until today (Scheme 15) [95,96,97,98]. It should be noted that the isolation of cyanocarbonimidoyl diazide (**77**) was also reported by Darzens in 1912 from the reaction of bromine cyanide (**76**) with sodium azide; however, the structure turned out to be wrong and was corrected by Hart in 1928 [99,100,101]. The geminal vinyl diazides **73**–**75** appeared in the literature between 1982 and 1991. Their synthesis was possible via simple substitution of a chloride with sodium azide in a mixture of acetone and water at temperatures between −20 °C and −15 °C. The diazide products were isolated by filtration (**73** and **74**) or direct separation of the water-insoluble liquid (for **75**). Methyl 3,3-diazido-2-cyanoacrylate (**73**) and ethyl 3,3-diazido-2-cyanoacrylate (**74**) were reported to be pale yellow to yellow-green crystalline solids, which are stable in solution at −15 °C for days, but decompose slowly at room temperature. At 70 °C and 50 °C, respectively, **73** and **74** explode spontaneously. Analytical confirmation of the structures was obtained from their infrared and NMR spectra as well as from mass spectrometry in the case of methyl 3,3-diazido-2-cyanoacrylate (**73**) [95,96,98,102]. 2-(Diazidomethylene)malononitrile (**75**) is described as an orange liquid. Infrared spectroscopy shows the azide stretching band at 2160 cm^−1^ [97]. Further analytical data for this compound are not available.

**Scheme 16:**
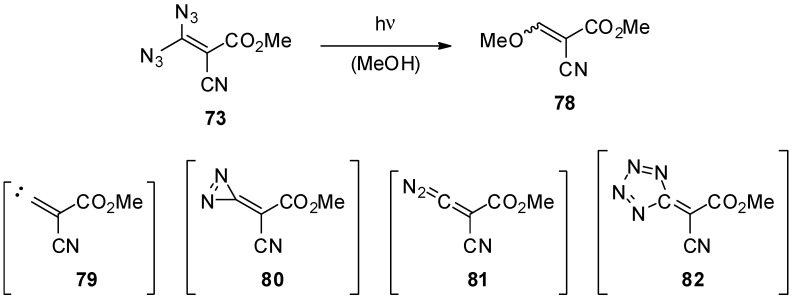
Photochemical reaction of methyl 3,3-diazido-2-cyanoacrylate (**73**).

The reactivity of the geminal vinyl diazides appears to be pretty versatile [103]. Unfortunately, several reports are, at least partially, contradictory, and we feel that some results are not free of doubt since analytical evidence is missing. Nevertheless, there are two fundamental reactivities of geminal vinyl diazides. The first one is the unimolecular thermal or photochemical decomposition. As shown in Scheme 16, irradiation of a methanolic solution of methyl 3,3-diazido-2-cyanoacrylate (**73**) gives a a low yield of methyl 2-cyano-3-methoxyacrylate (**78**) as a by-product (besides the thermal product **88** described below) [95,96,103]. Possible intermediates for this transformation are the free carbene **79**, the diazirine **80**, the diazoacrylate **81** or the tetrazole **82**.

**Scheme 17:**
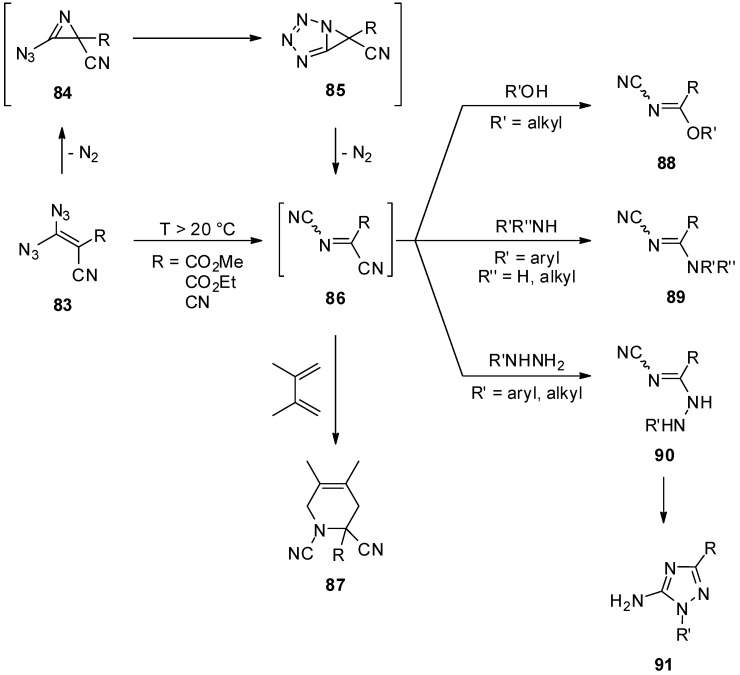
Thermal reactions of geminal vinyl diazides.

While the photochemical decomposition was not examined in further detail, the thermal reaction of geminal vinyl diazides is well-studied (Scheme 17) [95,96,97,98,103,104,105,106,107,108]. The key intermediate is the very electrophilic [109] double acceptor-substituted *N*-cyanoimine **86**. At temperatures over 20 °C, geminal vinyl diazides loose one molecule of nitrogen forming an azidoazirine **84**, which spontaneously cyclizes to the tetrazole **85** [108]. After loss of a second molecule of nitrogen and subsequent rearrangement, the *N*-cyanoimine **86** is generated. The *N*-cyanoimine **86** has been characterized in solution [97,108]; all isolation attempts led to polymerization [108,109]. The existence of this intermediate **86** was also proven by cycloaddition with 2,3-dimethylbuta-1,3-diene leading to the Diels-Alder product **87** [95,96,97,108,109]. In the presence of alcohols [95,96,108,109] or amines [104,105,106,107,108], the *N*-cyanoimine **86** is immediately attacked at the electrophilic carbon and, after elimination of hydrogen cyanide, isolable structures **88** and **89** are obtained in good yields. Reaction of the *N*-cyanoimine **86** with hydrazines leads to **90** in an analogous fashion. Further cyclisation of **90** then generates 1,2,4-triazoles **91** [98].

**Scheme 18:**
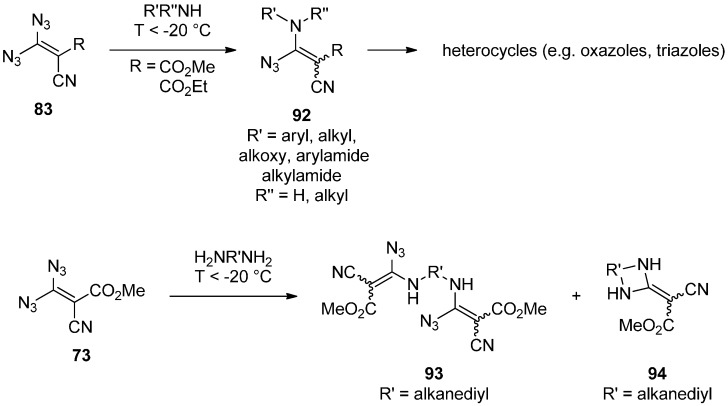
Nucleophilic substitution on geminal vinyl diazides.

The other fundamental reactivity of geminal vinyl diazides **83** is the nucleophilic substitution of an azide group. Formally, this must be seen as a 1,4-addition followed by elimination of hydrazoic acid. The resulting vinyl azides **92** can, in most cases, be isolated in good yields as crystalline solids [98,102,104,105,106,107,108,110,111]. Because of the thermal instability of geminal vinyl azides **83**, this reaction has to be performed at temperatures below −20 °C. Otherwise, the *N*-cyanoimine **86** (Scheme 17) would be generated before an intermolecular reaction can take place. As nucleophiles, primary [98,102,104,105,107,108,110,111] and secondary [102,107,111] amines, diamines [106,108,111] and hydrazines [98] were tested. With diamines, the substitution occurs either on two different molecules of the diazide **73** generating vinyl azides **93** [106,108,111] or on the same molecule delivering cyclic products **94** [106]. The reaction of simple amines with geminal vinyl diazides results in the substitution of one azide group, eventually affording vinyl azide **92** (Scheme 18). This vinyl azide species **92** was shown to undergo many different consecutive reactions giving access to a broad spectrum of nitrogen-containing heterocycles like tetrazolylidenes [98,106,108,110,111], imidazolylidenes [110,111], oxazoles [106,107,108,110], 1,2,3-triazoles [102,111], azirines [102], 2-methylidene-3,4,5,6-tetrahydropyrimidines [106] and 2-methylidene-2,3-dihydroperimidines [106]. The definite outcome of the reaction strongly depends on the substitution pattern of the vinyl azide **92** and the precise reaction conditions [98,102,103,106,107,108,110,111]. Nevertheless, geminal vinyl diazides are neglected but valuable starting materials for the synthesis of heterocycles with uncommon substitution patterns.

## 4. Geminal Triazides

**Scheme 19:**
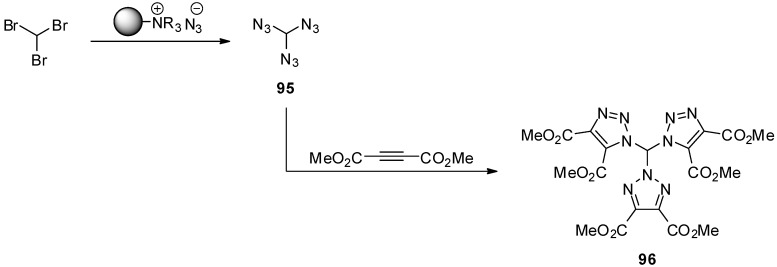
Synthesis of triazidomethane (**95**).

Triazidomethane (**95**), which was reported by Hassner *et al.* in 1990, is the archetype molecule of the triazido compound class [42]. Although it was never isolated in pure form due to its predicted volatility and hazardousness, the NMR-data and the cycloaddition reaction with dimethyl acetylenedicarboxylate giving the tristriazole product **96** confirm its structure. The synthesis of triazidomethane (**95**) was accomplished by mixing bromoform with a polymeric ammonium azide reagent, followed by washing of the resin with diethylether and concentration of the filtrate (Scheme 19). Triazidomethane (**95**) was obtained as a mixture together with diethylether and unreacted bromoform. So far, the physical properties of triazidomethane (**95**) are completely unknown. However, the authors indicated, that the transfer of this mixture into a NMR tube led to an explosion. Surprisingly, the cycloaddition with dimethyl acetylenedicarboxylate did not yield the expected tris(1-triazolyl) compound having three identical triazole moieties. Instead, the depicted tristriazole **96** with only two 1-triazolyl rings and one 2-triazolyl system was generated as evidenced by X-ray diffraction. It was assumed that, for steric reasons, the initially formed tris(1-triazolyl) compound rearranged to give **96**.

**Scheme 20:**
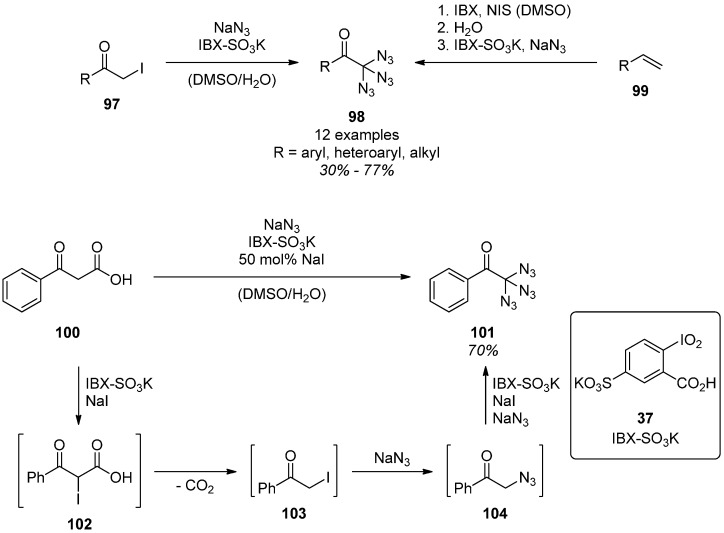
Triazide **98** synthesis with IBX-SO_3_K (**37**).

In 2014, another class of geminal triazides, the α,α,α-triazidocarbonyls **98**, was published by Kirsch *et al.* [59]. During the search for synthetic methods toward α-azidoesters, the α,α,α-triazidocarbonyls **98** were found as the main product in the azidation of 3-oxocarboxylic acids **100**. In related protocols, iodomethylketones **97** and terminal olefins **99** were employed as starting materials that were easier to handle (Scheme 20). All protocols have in common that IBX-SO_3_K (**37**) [62], the potassium salt of sulfonated 2-iodoxybenzoic acid (IBX), was used as oxidant and sodium azide as the azide source. The structure of the triazido products **98** was verified by ^15^N-NMR experiments and by triple cycloaddition with cyclooctyne. Based on preliminary experiments, the mechanism of the conversion of 3-oxocarboxylic acid **100** was assumed to start with the iodination of the enolizable position followed by decarboxylation. The resulting iodomethylketone **103** then undergoes substitution to furnish an azidomethylketone **104**. This cycle consisting of iodination and substitution is passed two more times to end up with the triazides **101**.

## 5. Tetraazidomethane

**Scheme 21:**
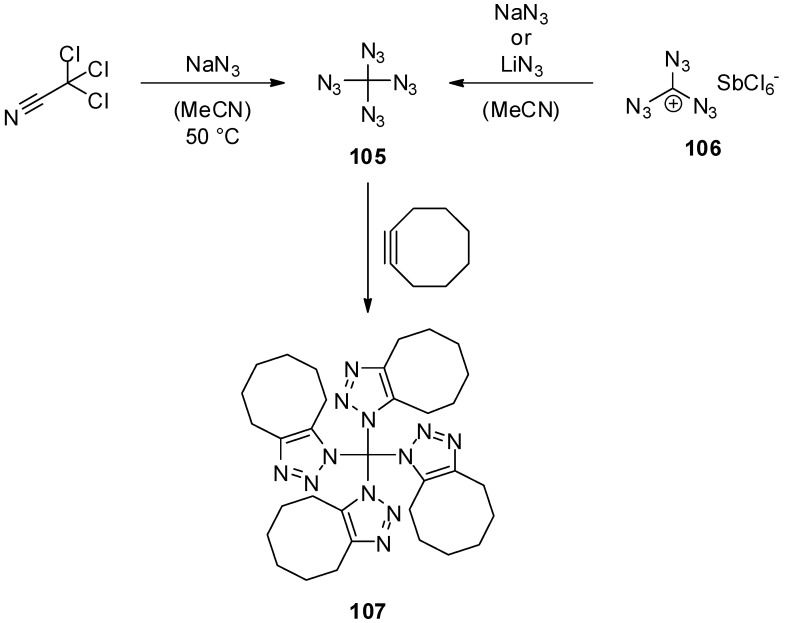
Synthesis of tetraazidomethane (**105**).

With a weight ratio of 93% nitrogen to 7% carbon, tetraazidomethane (**105**) may be the nitrogen-richest organic compound one could think of. The only report on this molecule was published in 2007 by Banert *et al.* [23]. In contrast to triazidomethane (**95**) [42], tetraazidomethane (**105**) is not available via direct substitution of carbon halides like tetrabromomethane. Instead, trichloroacetonitrile or alternatively triazidocarbenium hexachloroantimonate (**106**) were chosen as starting materials (Scheme 21). In the first case, treatment of trichloroacetonitrile with sodium azide in acetonitrile at 50 °C for 18 h yielded, after aqueous work up, tetraazidomethane (**105**), which was directly further converted with cyclooctyne. The cycloaddition product **107** was confirmed by X-ray diffraction. A pure sample of tetraazidomethane (**105**) could be isolated as a colorless liquid by preparative gas chromatography. It was fully characterized via IR, NMR, GCMS and HRMS.

**Scheme 22:**
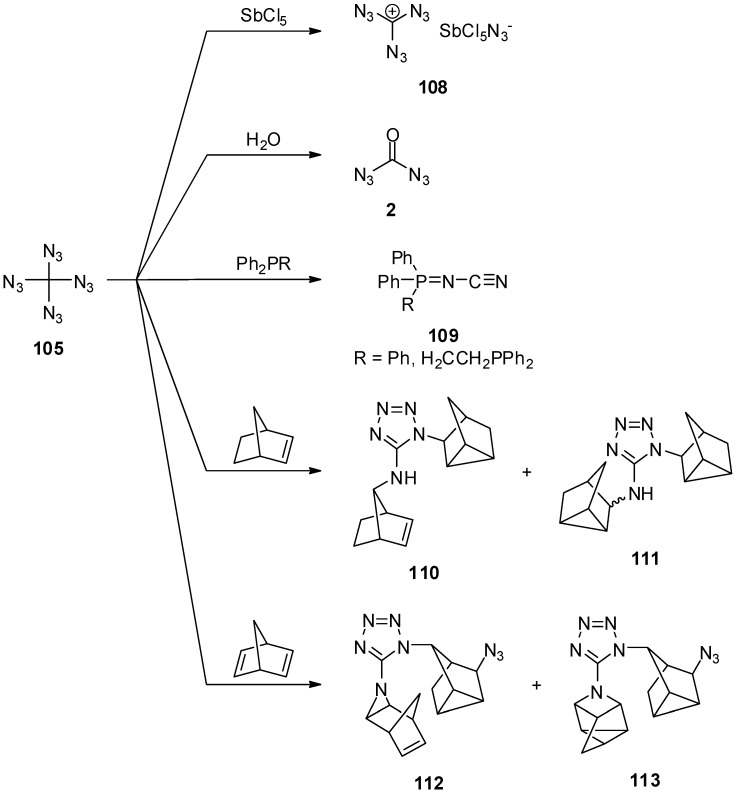
Reactivity of tetraazidomethane (**105**).

Tetraazidomethane (**105**) shows some expected and several unexpected reactivities besides the cycloaddition of cyclooctyne (Scheme 22). With Lewis acids like antimony pentachloride the triazidocarbenium salt **108** is formed. Under aqueous conditions carbonyl diazide (**2**) and two equivalents of hydrazoic acid are generated. More unexpected, the reaction of tetraazidomethane (**105**) with phosphines leads to the loss of ten nitrogen atoms, and the unusual cyanamide **109** is obtained as the product. With norbornene or norbornadiene, tetraazidomethane (**105**) does not undergo a simple cycloaddition. Instead, mixtures of aminotetrazoles **110**–**113** were isolated; these tetrazoles **110**–**113** are most likely generated in a multistep sequence. In view of these results, tetraazidomethane (**105**) is a very interesting compound with much research work left to explore the unique reactivity of this molecule.

## 6. Outlook

This review summarized the mentions of geminal di- and triazides in the literature. While easy methods for their preparation are available, not many researchers have had the courage to do chemistry with these compounds. Alternative methods for their synthesis and the elucidation of their reactivities are not nearly as established as one would expect for a compound class the early members of which have been known for more than 100 years. Hence, geminal organic diazides are, in principle, still a “young” class of molecules, and the lack of interest may have simply arisen from their possibly hazardous character, which cannot be denied. In particular, knowledge on their reactivities and synthetic potential seem to be notoriously underdeveloped. Despite a few reports, promising approaches to illuminate the chemical possibilities of geminal diazides have not been further pursued, in many cases. The same is true for geminal triazides, which have only been known for a short period of time, but are easily available, too. Besides the reported basic reactivities, the use of those compounds in high-energetic materials or for heterocycle synthesis remains to be further studied. We encourage researchers to step into this promising and stimulating field where is open space for new discoveries.
